# Spatiotemporal Evolution of Ebola Virus Disease at Sub-National Level during the 2014 West Africa Epidemic: Model Scrutiny and Data Meagreness

**DOI:** 10.1371/journal.pone.0147172

**Published:** 2016-01-15

**Authors:** Eva Santermans, Emmanuel Robesyn, Tapiwa Ganyani, Bertrand Sudre, Christel Faes, Chantal Quinten, Wim Van Bortel, Tom Haber, Thomas Kovac, Frank Van Reeth, Marco Testa, Niel Hens, Diamantis Plachouras

**Affiliations:** 1 Interuniversity Institute for Biostatistics and Statistical Bioinformatics, Hasselt University, Diepenbeek, Belgium; 2 European Centre for Disease Prevention and Control, Stockholm, Sweden; 3 Expertise centre for Digital Media, iMinds, tUL, Diepenbeek, Belgium; 4 Department of Public Health, University of Turin, Turin, Italy; 5 Centre for Health Economics Research and Modelling Infectious Diseases, Vaccine & Infectious Disease Institute, University of Antwerp, Antwerp, Belgium; Centro de Pesquisa Rene Rachou/Fundação Oswaldo Cruz (Fiocruz-Minas), BRAZIL

## Abstract

**Background:**

The Ebola outbreak in West Africa has infected at least 27,443 individuals and killed 11,207, based on data until 24 June, 2015, released by the World Health Organization (WHO). This outbreak has been characterised by extensive geographic spread across the affected countries Guinea, Liberia and Sierra Leone, and by localized hotspots within these countries. The rapid recognition and quantitative assessment of localised areas of higher transmission can inform the optimal deployment of public health resources.

**Methods:**

A variety of mathematical models have been used to estimate the evolution of this epidemic, and some have pointed out the importance of the spatial heterogeneity apparent from incidence maps. However, little is known about the district-level transmission. Given that many response decisions are taken at sub-national level, the current study aimed to investigate the spatial heterogeneity by using a different modelling framework, built on publicly available data at district level. Furthermore, we assessed whether this model could quantify the effect of intervention measures and provide predictions at a local level to guide public health action. We used a two-stage modelling approach: a) a flexible spatiotemporal growth model across all affected districts and b) a deterministic SEIR compartmental model per district whenever deemed appropriate.

**Findings:**

Our estimates show substantial differences in the evolution of the outbreak in the various regions of Guinea, Liberia and Sierra Leone, illustrating the importance of monitoring the outbreak at district level. We also provide an estimate of the time-dependent district-specific effective reproduction number, as a quantitative measure to compare transmission between different districts and give input for informed decisions on control measures and resource allocation. Prediction and assessing the impact of control measures proved to be difficult without more accurate data. In conclusion, this study provides us a useful tool at district level for public health, and illustrates the importance of collecting and sharing data.

## Introduction

The Ebola epidemic in West Africa was detected in March, 2014. On 8 August, 2014, WHO declared the event a Public Health Emergency of International Concern [1] and the UN General Assembly declared the epidemic a threat to global health and security [2]. On 9 May, 2015, Liberia was declared free of Ebola virus transmission but on 30 June, 2015, a new case was detected from an unknown chain of transmission [3,4]. In Guinea and Sierra Leone, the epidemic persists in a number of districts mainly between Conakry and Freetown [5]. As of 24 June, 2015, it has caused 27,443 probable, confirmed, and suspected cases of EVD in Guinea, Liberia and Sierra Leone, including 11,207 deaths [6].

A number of, mainly deterministic, SEIR transmission models (with Susceptible, Exposed, Infected, Recovered compartments) have been published that aimed to estimate epidemiological parameters, and to forecast the evolution of the epidemic [7–10]. Most models, and especially the ones early in the outbreak, were fitted on reported cumulative national data. Doing so, they did not account for the transmission heterogeneity of this outbreak and the serial correlation induced by the accumulation of data. However, in the course of the outbreak, others highlighted the importance of the spatial and temporal heterogeneity of the outbreak, questioning assumptions made by early models [11]. A study by King et al. [12] illustrated through simulations that deterministic models, fitted on cumulative incidence data, lead to substantial underestimation of the uncertainty in estimates and forecasts. In addition, fitting of the models was often done not taking into account the serial correlation. The clustered pattern of transmission could be attributed to variability in transmission settings (e.g. healthcare facilities, households, burials) [13], behaviour (e.g. expressions of mistrust) and control measures (e.g. contact tracing and monitoring and establishment of a treatment centre). However, there is still a lack of insight in the relative contribution of each factor to the transmission pattern [14].

A good understanding of the outbreak transmission may support an efficient allocation of resources at national and at district level. With our study, we aimed to develop a model that would overcome previously identified model limitations, including the not-used district level data and the assumption of homogenous transmission across districts. Our two-stage model is based on publicly available data that might improve the information for operational decisions to control the epidemic. The first stage is the use of a growth model that addresses the spatiotemporal correlation; the second stage is the use of a compartmental model–whenever deemed appropriate—that provides a district-specific estimation of the effective reproduction number–a composite dynamic estimate of the evolution of the outbreak—and its uncertainty. In addition, we performed a sensitivity analysis to study the effect of the model assumptions on the parameter estimates.

## Methods

### Data sources

#### Data on cases and deaths

We used publicly available district-level data on cumulative cases and deaths, reported from 30 December 2013 until 8 July 2015 through situational reports by the Ministries of Health of Guinea [15], Liberia [16] and Sierra Leone [17,18]. The data were collected and reported to the national authorities by the Ebola treatment units and diagnostic testing facilities in the three countries, following national guidelines and/or WHO case definitions [19].

Data were reported every two to three days, and more recently on a daily basis. The data sources provided no detailed information about the used case definition. Data for Liberia and Guinea were the reported total cumulative number of (suspected, probably and confirmed) cases and deaths, while for Sierra Leone, we calculated the sum of the reported suspected, probable and confirmed cases. This allowed us to calculate for each district the new cases and new deaths between two reporting intervals. The models were fitted to these new cases and new deaths over the corresponding time intervals and not to the cumulative data. The observed number of new cases and deaths were depicted by taking these new cases and new deaths and dividing them by the number of days the time intervals span.

A presentation of how the cases were reported can be found in S4 Fig. The reporting scheme for deaths was similar, but the dates at which reporting occurred is not necessarily the same.

#### Data on control measures

Publicly available situation reports of response measures were used to assess the intensity of interventions [20,21] (https://data.hdx.rwlabs.org/dataset?q=ebola). The publicly available data regarding interventions provided little detail and was not regular over time or over the entire outbreak region. Due to the complexity of response measures and limited availability of data, we used the presence of triage centres, holding or community care centres and Ebola Treatment Units (ETUs) as a surrogate marker of response activities.

### Models

#### Growth model

To compare growth patterns over time among districts, we used a flexible spatiotemporal growth rate model across all districts. This model allowed the estimation of the district-specific expected number of new cases per week, the district-specific time trend, the district-specific growth rate and the spatial distribution of the growth rate within the three countries. In addition, to investigate the effect of implemented intervention measures on the estimated growth rates, for each district a Pearson’s Chi-square test was used. Doing so, we tested, for different time lags, the association between positive and negative growth rates and the absence or presence of aforementioned intervention measures. We used Integrated Nested Laplace Approximation [22] as a more flexible estimation method and alternative for the more computationally intensive Markov chain Monte Carlo (MCMC). We made a growth rate distribution heat map as a method to visualise the weekly change for each district-specific rate of infection with an overlay of intervention measures.

#### Compartmental model

Further, district-specific SEIR compartmental models were fitted to the number of newly reported cases and deaths (see S1 File for more details). We applied this model to data of several districts to address within-district disease evolution over time. In this paper we show the obtained results for a selection of rural and urban districts: Forecariah (Guinea), Conakry (Guinea), Western Area Urban (Sierra Leone), and Grand Cape Mount (Liberia). This selection was based on events of interest during the course of the outbreak e.g. sudden increase in cases. We were, however, also restricted by inconsistencies in the data as pointed out as a limitation of the model in the discussion. For each of these four districts the effective reproduction number, Re(t), was estimated over time. The SEIR model incorporates disease-related mortality by making the distinction between survivors and non-survivors. It also takes into account an underreporting factor for cases and deaths. Goodness of fit was assessed visually.

We assessed retrospectively the quality of three-week long predictions made at 4 different time points, for the selected districts and we compared these predictions with the actual observed number of cases and deaths.

#### Assumptions and sensitivity analysis

For the compartmental models, we used prior estimates of the incubation period for EVD (9·4 days), the duration of infectiousness for survivors (16·4 days) and deceased (7·5 days) [23]. The reproductive number was modelled with a piecewise constant interval of 21 days. The remaining parameters are estimated via an MCMC approach. The MCMC procedure, which we made publicly available, was performed in R 3.1.1 using the Laplaces-Demon package [24,25].

Furthermore, due to reclassification of suspected cases over time, the cumulative data–expected to increase monotonically over time, decreased at certain time points. It was thus necessary to monotonize the data. The algorithm that was used to do so is described in S1 File.

Lastly, we assessed the sensitivity of our results to the model assumptions by performing a sensitivity analysis. We investigated the estimability of the fixed parameters mentioned in the previous paragraph, the effect of the number of exposed individuals at time 0, transmission through contacts with bodies of dead people, and protective immunity by asymptomatic infections. We compared the models with Deviance Information Criterion (DIC).

More details about the models and estimation methods, as well as results from the sensitivity analysis can be found in the S1 File. Our compiled data and code are made available for reproducibility purpose.

## Results

**Fig 1:**
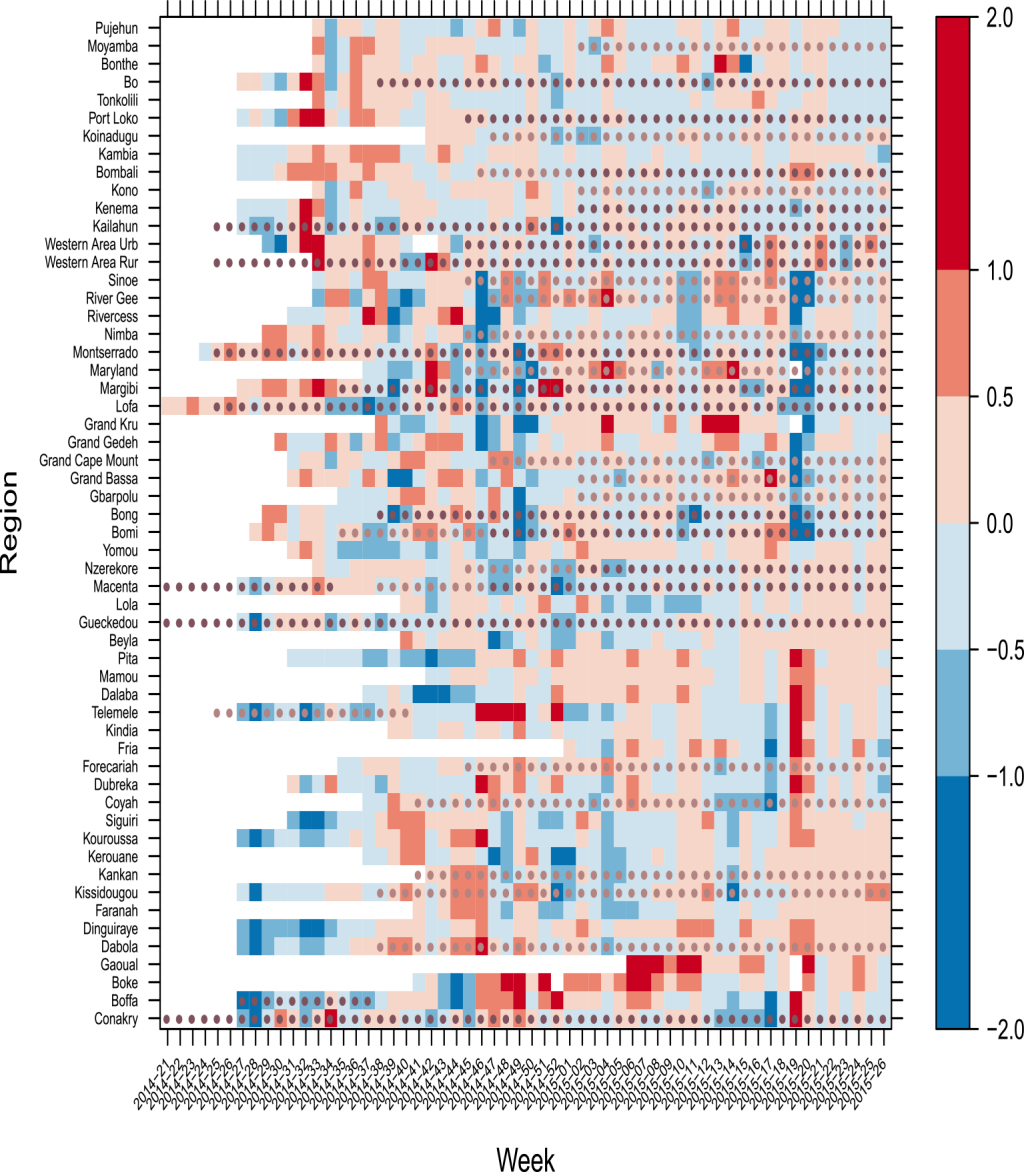
Estimated weekly growth rates per district and implemented intervention measures for Guinea, Sierra Leone and Liberia, 2014–2015. Red colours indicate an increase in number of weekly cases, whereas blue colours indicate a decline. Periods for which no reported cases are available are shown in white. A light dot indicates that a triage, holding centre or CCC is in place and a dark dot indicates that an ETU or ETU and CCC are in place.

Results of the growth rate model are shown in the heat map in Fig 1. Comparing the growth rates in the different districts (rows), it was clear that the outbreak did not evolve uniformly over districts. Pearson’s Chi-square test for decrease in growth rate after implementation of control measures, for different time lags, did not reveal any insights (results not shown). A map of the geographical distribution of the estimated growth rates can be found in S3 Fig.

**Fig 2:**
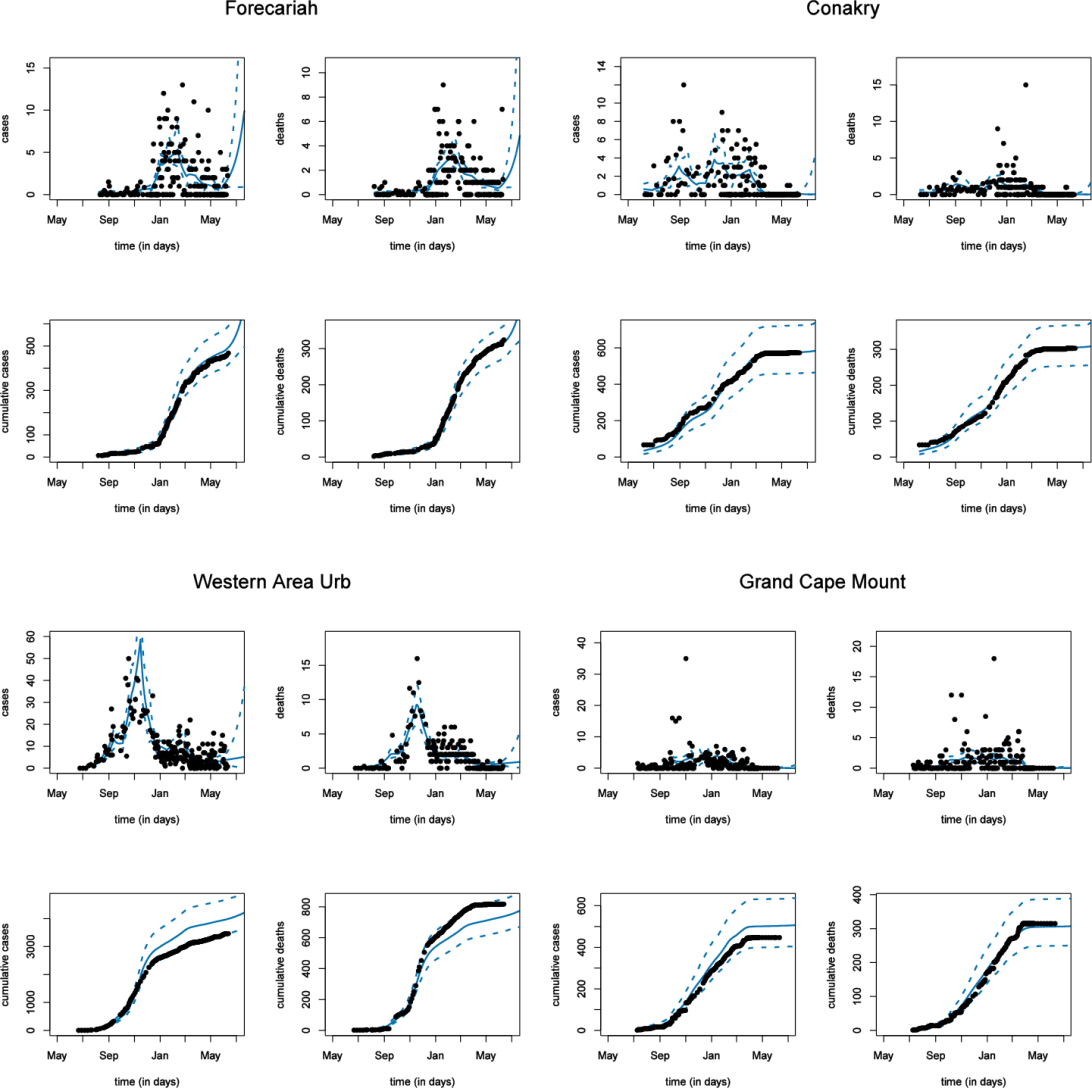
Observed (black) and estimated (blue) number of new cases (top left), new deaths (top right), cumulative cases (bottom left) and cumulative deaths (bottom right) per district. Dashed lines are 95% credible intervals.

**Fig 3:**
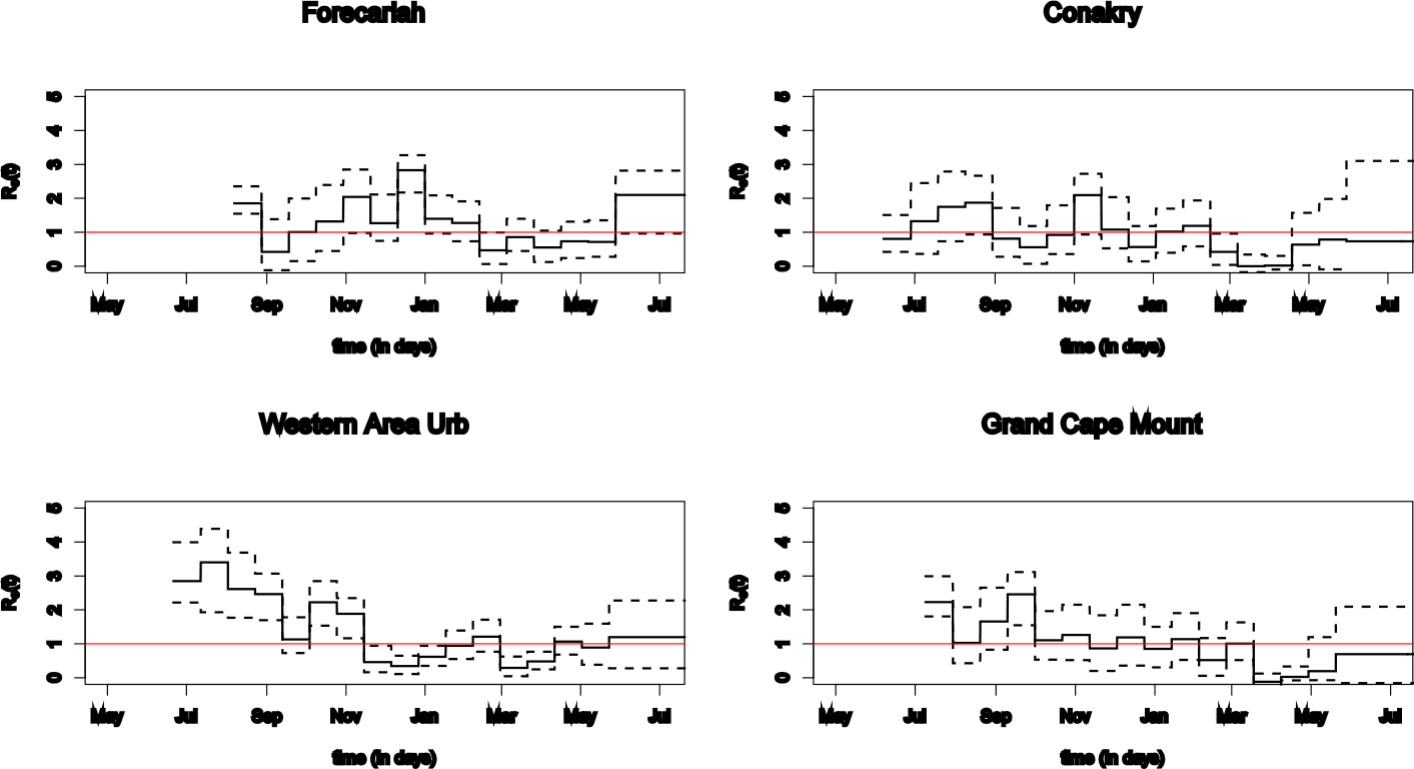
Estimated reproduction number per district with 95% posterior intervals. The threshold value of one is indicated by a red horizontal line.

**Fig 4:**
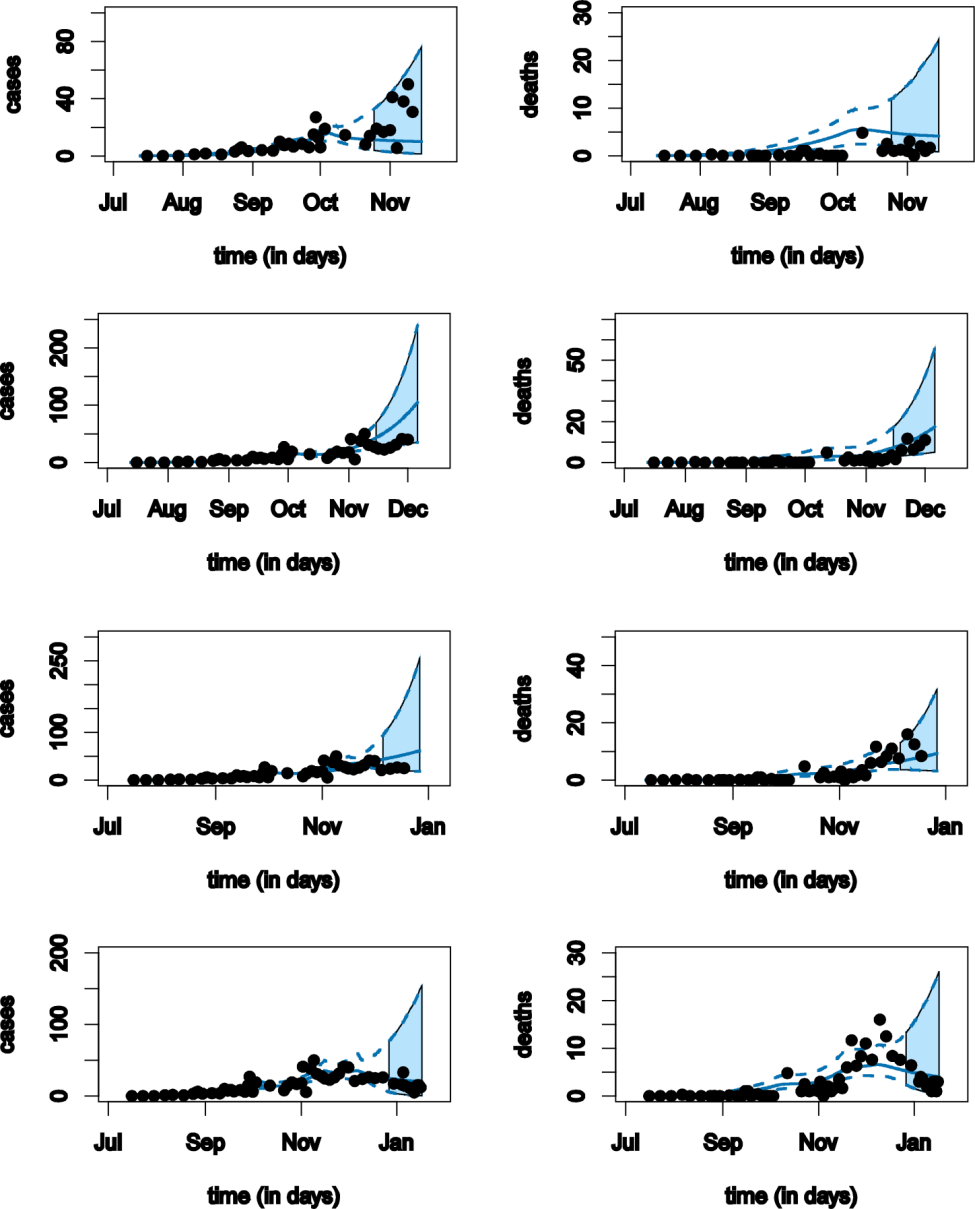
Three-week prediction of new cases (left) and deaths (right) for Western Area Urban at 24 October, 14 November, 5 December and 26 December 2014 (top to bottom). Light blue regions are the predicted time periods and estimation is based on all data before that time point.

Results of the SEIR models for the four selected districts are presented in Figs 2–4. The observed and estimated number of new and cumulative cases and deaths are shown in Fig 2. From this figure, we observe that the model fits both the number of cases and the number of deaths relatively well. Also at the level of the cumulative numbers model and data show a reasonable fit.

The estimated effective reproduction numbers over time are shown in Fig 3. Re(t) ranges from below unity to up to 3·5. Furthermore, estimates are below one for all four districts in the last time period. However, the 95% credible intervals indicate substantial variability.

Results of the short-term predictions are presented in Fig 4 for Western Area Urban. Note that the credible intervals do not contain all data points. Hence, even within a 3-week forecast period, the models are not always able to capture all the trends.

The results of the sensitivity analysis (S1 File) show that the fixed parameters in our model are not estimable from the data. Further, taking into account the transmission from dead bodies does not improve model fit. We do, however, see an improvement when including an increasing (from 10 to 40%) proportion of asymptomatic cases (S4 Table).

## Discussion

The results of our study strengthen the evidence of a strong temporal and spatial variability of the EVD transmission at a subnational level in the affected regions of Guinea, Sierra Leone and Liberia. The variable transmission dynamics are a major challenge for the implementation of intervention measures and the mobilisation of resources among districts. This complexity highlights the importance of constant monitoring and the usefulness of quantitative tools, thereby taking full account of the uncertainty, to inform the response.

Our growth model quantifies spatiotemporal transmission patterns at a sub-national level, which cannot be derived from visual inspection of incidence curves and maps alone. The visualisation of the growth rates with a two dimensional (time and space) heatmap, is useful for decision makers to make evidence based informed decisions on resource allocation. On the other hand, our compartmental model allows the calculation of a quantitative measure of transmission, Re(t), that can be used to compare and communicate about differences in outbreak dynamics between districts and over time.

The combined model illustrates how district-level data can be used to gain a quantitative insight in the complex outbreak dynamics. Both models show how the trend varies widely among the districts and changes quickly in time and space (Figs 1 and 3). While our estimates of Re(t) are within the range of published estimates, most of the published estimates were derived from country-level data and do not provide the granularity we provide at time-dependent district level. The wide range of Re(t) between near 0 and 3·5 illustrates the need to complement national with district data driven models, to support public health action.

We further show that it is difficult to generate accurate predictions. Forecast results should be interpreted with caution, as control measures and behavioural changes cannot be sufficiently quantified with the publicly available data. Also, there are still gaps in our basic knowledge about the disease spread that could potentially explain outliers, departing from modelling approaches. We think here, for example, of the three last reported cases in Liberia; one from suspected sexual transmission months after the source case recovered from disease [26], and most recently two connected cases without any recognised link to outbreak chains.

One of the limitations of our model is the assumption of constant underreporting. Previous studies have also assumed a proportion of underreporting [13]. Knowledge about the level and changes in underreporting over time would improve the estimates of transmission dynamics. Unfortunately we do not have data to assess the magnitude or the variability of underreporting. Also, inconsistent reporting with undocumented backlogging and the absence of dates of disease onset may affect the accuracy of the estimates and need to be taken into consideration when interpreting the results [27]. Furthermore, the district-specific SEIR model is a mathematical model assuming a deterministic disease process. As a consequence, the second phase of our approach was deemed inappropriate for some districts, because the data didn’t seem to follow any consistent pattern, presumably due to the aforementioned inconsistencies in detection and reporting and the sporadic introduction of cases.

EVD can be transmitted through contact with dead bodies; therefore, a model accounting for this transmission was included in the sensitivity analysis. However, this model did not improve the fit to the data. Most likely, the extent to which dead bodies versus cases contribute to transmission is indistinguishable with this model and requires more information and a fully stochastic modelling approach on disaggregated data, which is not publicly available.

Since there is evidence suggesting the presence of asymptomatic Ebola infections [28], we looked at the effect of accounting for protective immunity by asymptomatic infection. We observed that the model fit improved with increasing proportion of asymptomatic cases, suggesting that our data do not reject the hypothetical occurrence of asymptomatic cases. Asymptomatic cases could partially explain why the epidemic did not reach the expected incidence as predicted by models ignoring them. This again highlights the need for serological studies in order to clarify the role of asymptomatic infection.

While our sensitivity analysis assesses the influence of unknown parameters, it cannot substitute for non-public data. The growth rate and compartmental models can be run in real time using our published code and dataset, and can be improved by organizations that have additional data available or to explore adaptations to the models and parameters. In the end, different modelling approaches bring different insights and will improve our ability to effectively support public health action. We recommend that minimal datasets and standards for data processing, including de-identification, and data sharing will be developed for future multi-country outbreaks, especially Public Health Events of International Concern under the International Health Regulations. The importance of this has also been retained as a conclusion in a recent research paper on this topic [29].

Our two-stage modelling approach, built with the most detailed publicly available data, provides time-dependent district-specific quantitative measures of growth and transmission. We hope that such tool, in addition to other approaches, can complement public health action against such devastating events as the West-African Ebola epidemic.

## Supporting Information

S1 Fig Cumulative cases per district and implemented intervention measures. A light dot indicates that a triage, holding centre or CCC is in place and a dark dot indicates that an ETU or ETU and CCC are in place.
(JPEG)

S2 Fig Cumulative deaths per district and implemented intervention measures. A light dot indicates that a triage, holding centre or CCC is in place and a dark dot indicates that an ETU or ETU and CCC are in place.
(JPEG)

S3 Fig Estimated growth rate per district and implemented intervention measures during week 21 and 40 of 2014 and week 8 and 26 of 2015. ‘1’ triage, holding centre or CCC is in place; ‘2’ ETU or ETU plus CCC is in place.
(PDF)

S4 Fig Flow diagram for the SEIR model with distinction between cases that survive and fatal cases. (JPG)

S5 Fig Schematic representation of reporting of case notifications. (JPG)

S1 File Supplementary file. (DOCX)

S1 Table Prior distributions. (PDF)

S2 Table Parameter estimates with 95% posterior confidence intervals. Note that for Conakry a ***U*(0, 100)** prior for E(0) was used.
(PDF)

S3 Table Parameter estimates sensitivity analysis. Fixed values are indicated in bold, blue values indicate model differences compared to the final model 1.
(PDF)

S4 Table Parameter estimates sensitivity analysis. Fixed values are indicated in bold, blue values indicate changes compared to the final model 1.
(PDF)

## References

[1] Hawkes N. Ebola outbreak is a public health emergency of international concern, WHO warns. BMJ. 2014; 349: g5089 10.1136/bmj.g5089 25106874

[2] United Nations Security Council. Resolution 2177 (2014). Adopted by the Security Council at its 7268th meeting on 18 September 2014. Available: http://www.un.org/en/ga/search/view_doc.asp?symbol=S/RES/2177 (2014)

[3] World Health Organization. The Ebola outbreak in Liberia is over. 2015 May 9. Available: http://www.who.int/mediacentre/news/statements/2015/liberia-ends-ebola/en/.

[4] World Health Organization. Ebola Situation Report—24 June 2015. Available: http://apps.who.int/ebola/current-situation/ebola-situation-report-24-june-2015 Accessed 2 Jul 2015.

[5] UN Mission for Ebola Emergency Response (UNMEER). Available: https://ebolaresponse.un.org/un-mission-ebola-emergency-response-unmeer.Accessed 1 Jul 2015.

[6] World Health Organization. Ebola Situation Report—18 March 2015. Available: http://apps.who.int/ebola/current-situation/ebola-situation-report-18-march-2015.Accessed 25 March 2015.

[7] Althaus CL. Estimating the reproduction number of Ebola Virus (EBOV) during the 2014 outbreak in West Africa. PLOS Currents Outbreaks, 2014 9 2 Edition 1. 10.1371/currents.outbreaks.91afb5e0f279e7f29e7056095255b288 PMC4169395 25642364

[8] Fisman D, Khoo E, Tuite A. Early Epidemic Dynamics of the West African 2014 Ebola Outbreak: Estimates Derived with a Simple Two-Parameter Model. PLOS Currents Outbreaks. 2014 9 8 Edition 1. 10.1371/currents.outbreaks.89c0d3783f36958d96ebbae97348d571 PMC4169344 25642358

[9] Gomes MFC, Pastore y Piontti A, Rossi L, Chao D, Longini I, Halloran ME, et al Assessing the international spreading risk associated with the 2014 West African Ebola outbreak. PLOS Currents Outbreaks. 2014 9 2 Edition 1. 10.1371/currents.outbreaks.cd818f63d40e24aef769dda7df9e0da5 PMC4169359 25642360

[10] Nishiura H, Chowell G. Early transmission dynamics of Ebola virus disease (EVD), West Africa, March to August 2014. Euro Surveill. 2014; 19(36): pii:20894. 10.2807/1560-7917.ES2014.19.36.20894 25232919

[11] Chowell G, Viboud C, Hyman JM, Simonsen L. The Western Africa ebola virus disease epidemic exhibits both global exponential and local polynomial growth rates. PLOS Currents Outbreaks. 2015 1 21 Edition 1. 10.1371/currents.outbreaks.8b55f4bad99ac5c5db3663e916803261 PMC4322058 25685633

[12] King AA, Domenech de Celles M, Magpantay FM, Rohani P. Avoidable errors in the modelling of outbreaks of emerging pathogens, with special reference to Ebola. Proc. R. Soc. B. 2015; 282(20150347). 10.1098/rspb.2015.0347. PMC4426634 25833863

[13] Merler S, Ajelli M, Fumanelli L, Gomes MFC, Pastore y Piontti A, Rossi L, et al Spatiotemporal spread of the 2014 outbreak of Ebola virus disease in Liberia and the effectiveness of non-pharmaceutical interventions: a computational modelling analysis. The Lancet Infectious Diseases. 2015; 15(2): 204–11. 10.1016/S1473-3099(14)71074-6 25575618 PMC4409131

[14] Chowell G, Simonsen L, Viboud C, Kuang Y. Is West Africa Approaching a Catastrophic Phase or is the 2014 Ebola Epidemic Slowing Down? Different Models Yield Different Answers for Liberia. PLOS Currents Outbreaks. 2014 11 20 Edition 1. 10.1371/currents.outbreaks.b4690859d91684da963dc40e00f3da81 PMC4318911 25685615

[15] Nations U. West Africa: Ebola Outbreak 2014–2015. Available: http://www.humanitarianresponse.info/disaster/ep-2014-000041-gin/documents%20and%20since%20April%202015%20http://guinea-ebov.github.io/sitreps.html. Accessed 1 Jul 2015.

[16] Liberia MoHaSWRo. Facts about Ebola Virus Disease. Available: http://www.mohsw.gov.lr/content_display.php?submenu_id=72&sub=submenu. Accessed 1 Jul 2015.

[17] Sanitation Moha. Ebola Situation Report. Available: http://health.gov.sl/?page_id=583. Accessed 1 Jul 2015.

[18] National Ebola Response Centre (NERC). EVD Daily MoHS Update. 2014–2015. Available: http://nerc.sl/. Accessed 1 Jul 2015.

[19] World Health Organization. Case definition recommendations for Ebola or Marburg Virus Diseases. 9 August 2014. Available: http://www.who.int/csr/resources/publications/ebola/ebola-case-definition-contact-en.pdf. Accessed 16 January 2015.

[20] Rapport De La Situation Epidemiologique | EPI Situation Report. Available: http://www.humanitarianresponse.info/en/operations/west-and-central-africa/documents/disasters/33204. Accessed 1 Jul 2015.

[21] Exchange HD. West Africa: Ebola Outbreak. Available: https://data.hdx.rwlabs.org/ebola. Accessed 1 Jul 2015.

[22] Rue H, Martino S, Chopin N. Approximate Bayesian inference for latent Gaussian models by using integrated nested Laplace approximations. Journal of the Royal Statistical Society: Series B (Statistical Methodology). 2009; 71(2): 319–92.

[23] Lewnard JA, Ndeffo Mbah ML, Alfaro-Murillo JA, Altice FL, Bawo L, Nyenswah TG, et al Dynamics and control of Ebola virus transmission in Montserrado, Liberia: a mathematical modelling analysis. The Lancet Infectious Diseases. 2014; 14(12): 1189–1195. 10.1016/S1473-3099(14)70995-8 25455986 PMC4316822

[24] Rosenthal JS. AMCMC: An R interface for adaptive MCMC. Computational Statistics & Data Analysis. 2007; 51(12): 5467–70.

[25] Roberts GO, Rosenthal JS. Examples of Adaptive MCMC. Journal of Computational and Graphical Statistics. 2009; 18(2): 349–67.

[26] Christie A, Davies-Wayne GJ, Cordier-Lasalle T, Blackley DJ, Scott Laney A, Williams DE, et al Possible Sexual Transmission of Ebola Virus—Liberia, 2015. Morbidity and Mortality Weekly Report. 2015; 64: 479–81. 25950255 PMC4584553

[27] Azmon A, Faes C, Hens N. On the estimation of the reproduction number based on misreported epidemic data. Stat Med. 2014; 33(7): 1176–92. 10.1002/sim.6015 24122943

[28] Bellan SE, Pulliam JRC, Dushoff J, Meyers LA. Ebola control: effect of asymptomatic infection and acquired immunity. The Lancet. 2014; 384(9953): 1499–500. 10.1016/S0140-6736(14)61839-0 PMC4829342 25390569

[29] Sane J, Edelstein M. Centre on Global Health Security Overcoming barriers to data sharing in public health: A global perspective. London: The Royal Institute of International Affairs, 2015 Available: https://www.chathamhouse.org/sites/files/chathamhouse/field/field_document/20150417OvercomingBarriersDataSharingPublicHealthSaneEdelstein.pdf.

