# Supplementary figures and images for: Spatiotemporal Evolution of Ebola Virus Disease at Sub-National Level during the 2014 West Africa Epidemic: Model Scrutiny and Data Meagreness
Source: PLoS One. 2016 Jan 15;11(1):e0147172. doi: 10.1371/journal.pone.0147172 (PMC4714854; doi:10.1371/journal.pone.0147172)

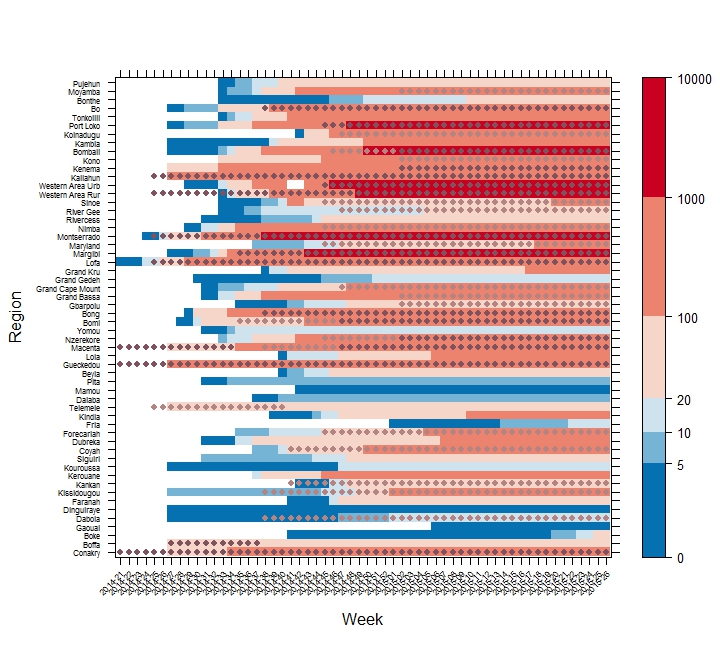

Supplement: S1 Fig — A light dot indicates that a triage, holding centre or CCC is in place and a dark dot indicates that an ETU or ETU and CCC are in place. (JPEG) [file pone.0147172.s001.jpeg]

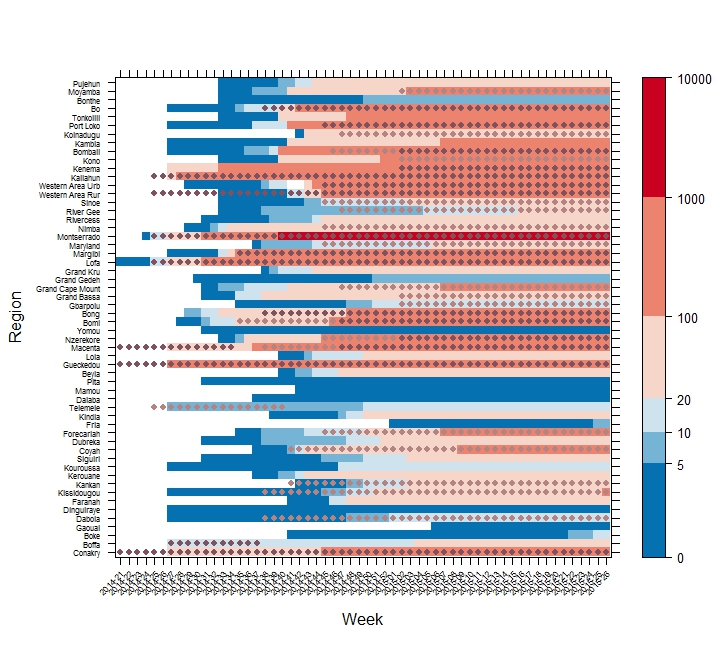

Supplement: S2 Fig — A light dot indicates that a triage, holding centre or CCC is in place and a dark dot indicates that an ETU or ETU and CCC are in place. (JPEG) [file pone.0147172.s002.jpeg]

Week  
2014-21

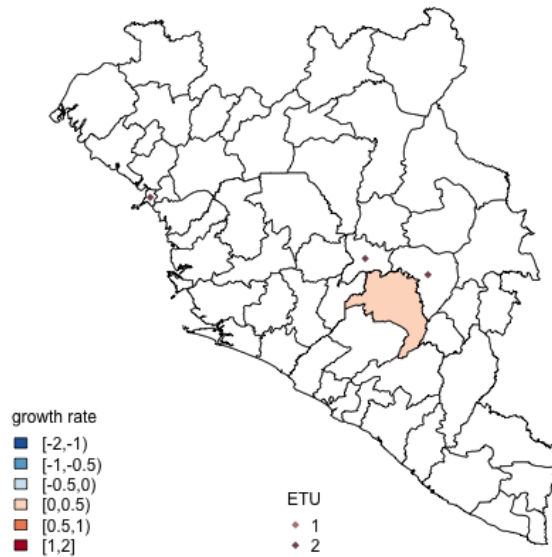

Week  
2014-40

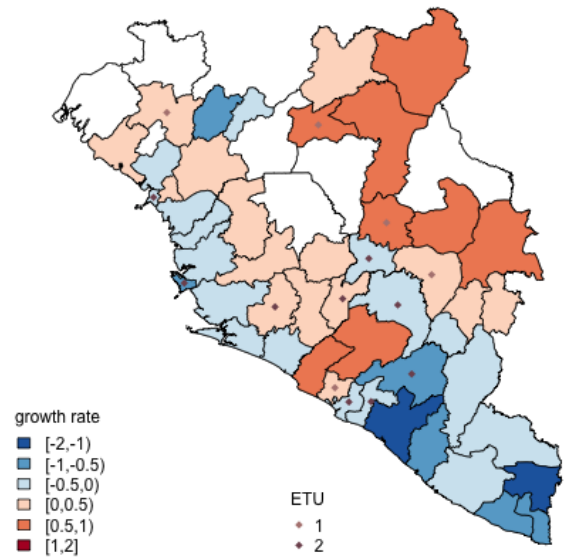

Week  
2015-08

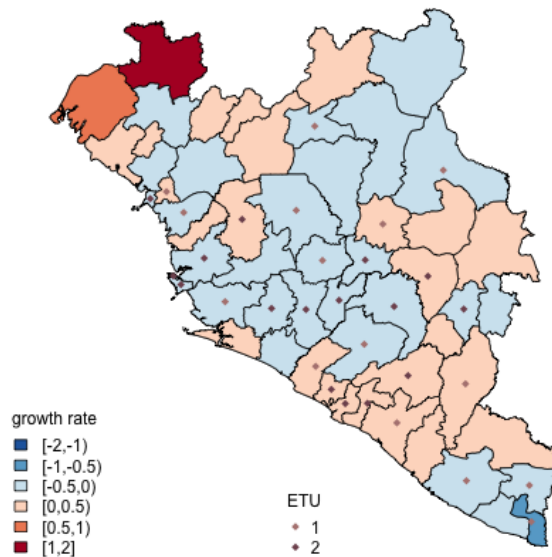

Week  
2015-26

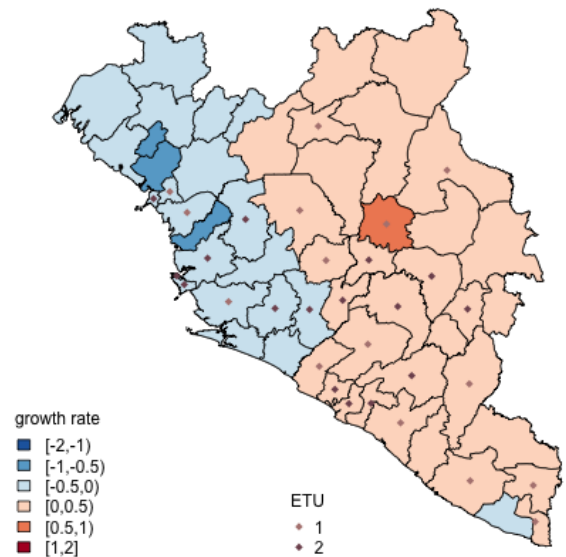

Supplement: S3 Fig — ‘1’ triage, holding centre or CCC is in place; ‘2’ ETU or ETU plus CCC is in place. (PDF) [file pone.0147172.s003.pdf]

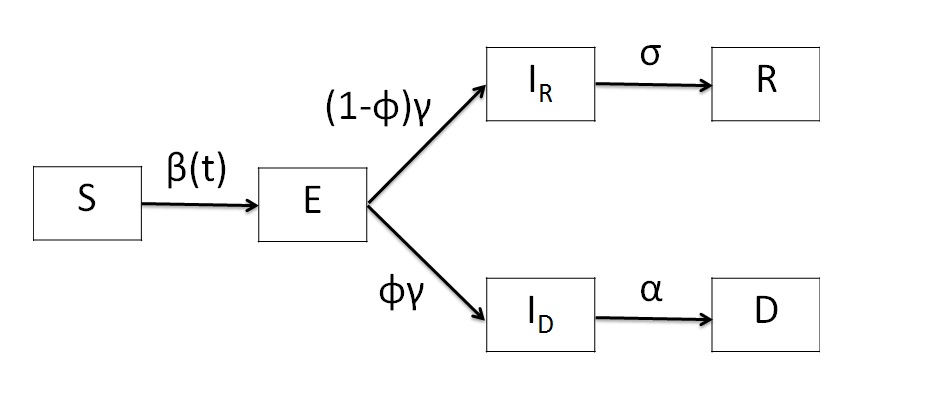

Supplement: S4 Fig — (JPG) [file pone.0147172.s004.jpg]

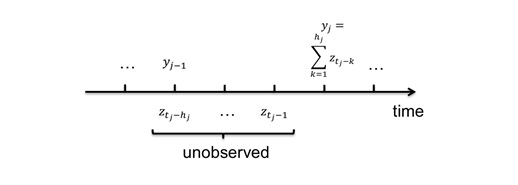

Supplement: S5 Fig — (JPG) [file pone.0147172.s005.jpg]
