# Supplementary material for: Spatiotemporal Evolution of Ebola Virus Disease at Sub-National Level during the 2014 West Africa Epidemic: Model Scrutiny and Data Meagreness
Source: PLoS One. 2016 Jan 15;11(1):e0147172. doi: 10.1371/journal.pone.0147172 (PMC4714854; doi:10.1371/journal.pone.0147172)
Supplement: S1 File — (DOCX) [file pone.0147172.s006.docx]

## Supplementary Material

**Spatiotemporal growth rate model**

We use a statistical model that describes the spatiotemporal growth rate. The weekly number of new infections in each district is modeled via a count-distribution allowing for possible overdispersion. The expected number of cases is modeled using a spatiotemporal function that makes a distinction between the temporal process and the spatial process. While the number of cases in a district is allowed to depend on the number of cases in this district the week before, the number of cases also depend on the number of cases in the neighboring districts. The growth rate is obtained numerically as the derivative of the expected number of cases. This model can be written as :

$$I_{i}\left( t \right)\sim NegBin\left( \mu_{i}\left( t \right) \right),$$

$$\log\left( \mu_{i}(t) \right)=\beta_{0}+\beta C_{i}+f_{i}\left( t \right),$$

in which

- $I_{i}\left( t \right)$ is the number of newly infected cases in week *t* and district *i*;
- $C_{i}$ is the indicator variable for district *i*;
- $f_{i}(t)$ is a spatio-temporal function.

The function $f_{i}\left( t \right)$ is defined as a separable spatio-temporal model using a spatially-correlated autoregressive AR(1) process:

$$f_{i}\left( t \right)=x_{i,t}=\phi x_{i,t-1}+\epsilon_{i,t},$$

with $\phi$ a scalar, and $\epsilon_{i,t}$ a Gaussian spatial Random Walk Process. The latter is defined as

$$\epsilon_{i,t}\sim N\left( \frac{1}{n_{i}}\sum_{i\sim j} \epsilon_{j,t},\frac{1}{n_{i}\tau} \right),$$

where $n_{i}$ is the number of neighbors of district *i*, and $i\sim j$ indicates that districts *i* and *j* are neighboring districts.

While Markov Chain Monte Carle (MCMC) methods are often used to estimate the parameters of interest in this model, it is computationally intensive. Therefore, we use Integrated Nested Laplace Approximation (INLA; Rue, Martino and Chopin, 2009) as an alternative estimation method. The INLA approach is a fast Bayesian inference tool that uses accurate approximations to the densities of the hyperparameters and latent variables in the model.

This spatiotemporal model allows estimating, amongst others:

- the area-specific expected number of new cases per week

$$\exp\left( \hat{\beta_{0}}+\hat{\beta}C_{i}+\hat{f_{i}}\left( t \right) \right)$$

- the area-specific time trend $\hat{f_{i}}\left( t \right)$
- the area-specific growth rate $\frac{\hat{{df}_{i}}(t)}{dt}$, which is estimated as $\frac{{x_{i,t+1}-x}_{i,t-1}}{2}$ for week *t*
- the spatial distribution of the growth rate.

In order to take into account reporting spikes at some of the weeks, dummy covariates are added to the model, corresponding to these weeks.

**Cumulative number of cases and deaths**

To complement the heat map of the estimated growth rates in Figure 1 in the main text, the cumulative number of cases and deaths are shown in Figures S1 and S2, respectively. These figures make it possible to identify the most affected regions.


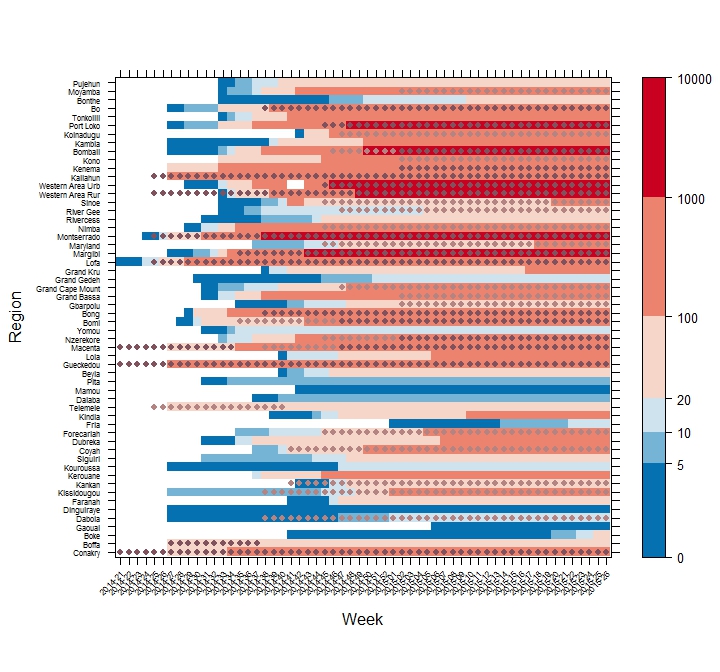


Figure S1: Cumulative cases per district and implemented intervention measures. A light dot indicates that a triage, holding centre or CCC is in place and a dark dot indicates that an ETU or ETU and CCC are in place.


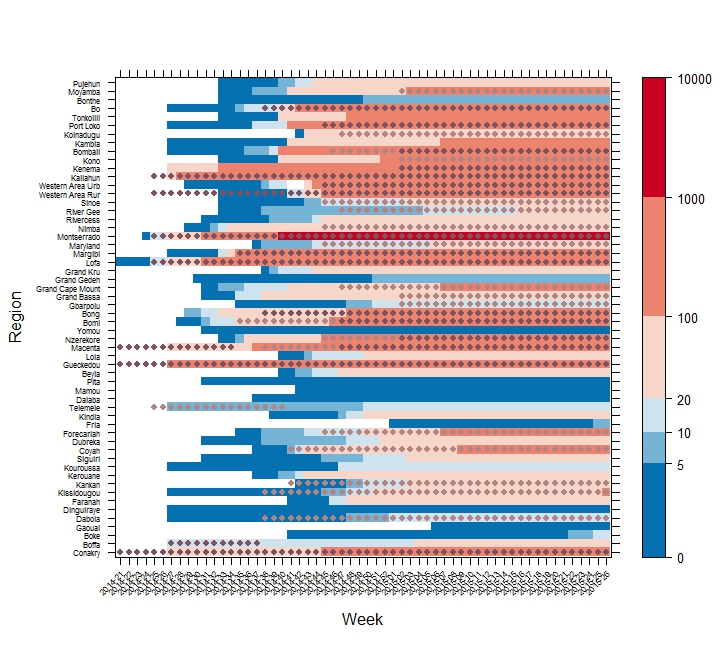


Figure S2: Cumulative deaths per district and implemented intervention measures. A light dot indicates that a triage, holding centre or CCC is in place and a dark dot indicates that an ETU or ETU and CCC are in place.

**Estimated growth rates**

Figure S3 shows the estimated growth rates and implemented intervention measures for four selected time points on a geographical map of West Africa. This figure emphasizes the spatial heterogeneity of the outbreak, even within countries. Movies showing the evolution of the spatiotemporal rates are available at www.simid.be.


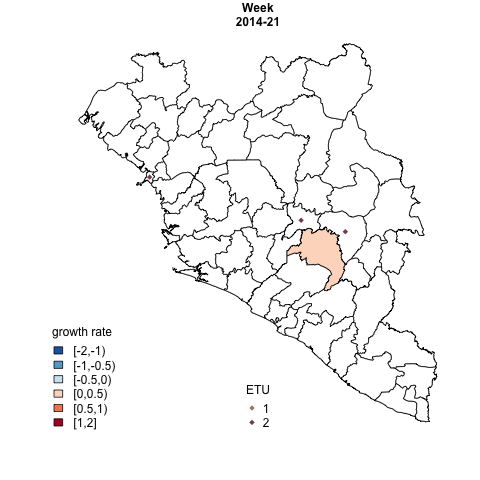

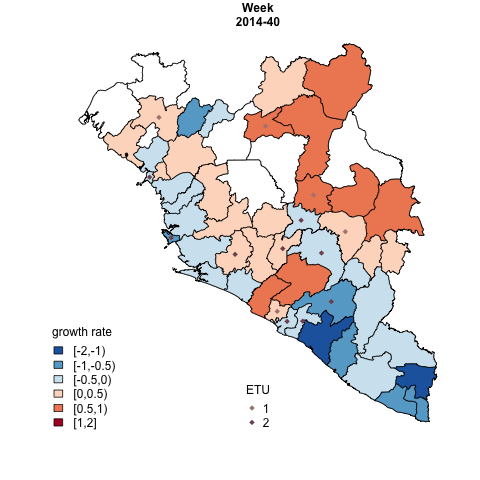

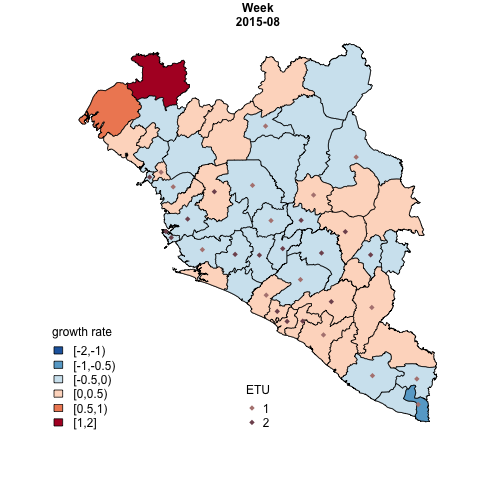

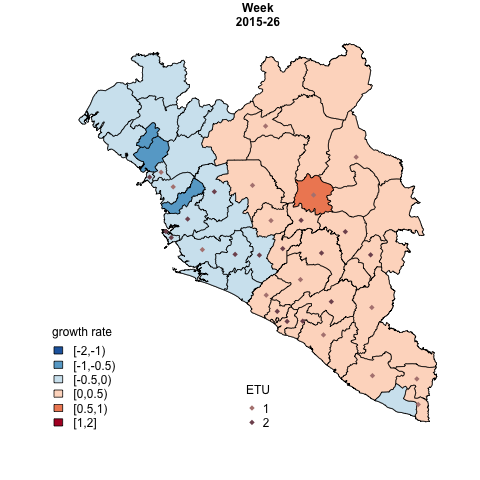


**Figure S3:** Estimated growth rate per district and implemented intervention measures
during week 21 and 40 of 2014 and week 8 and 26 of 2015. ‘1’ triage, holding centre or CCC is in place; ‘2’ ETU or ETU plus CCC is in place.

**SEIR compartmental model**

**Model**

We use a deterministic model which describes the disease dynamics in a selected district by partitioning the population into different disease states or compartments. The flow between disease states is typically represented by a set of (partial) differential equations. We use a version of a Susceptible-Exposed-Infected-Recovered (SEIR) compartmental model that makes the distinction between cases that survive and cases that die. This model is depicted as a flow diagram in Figure S4.


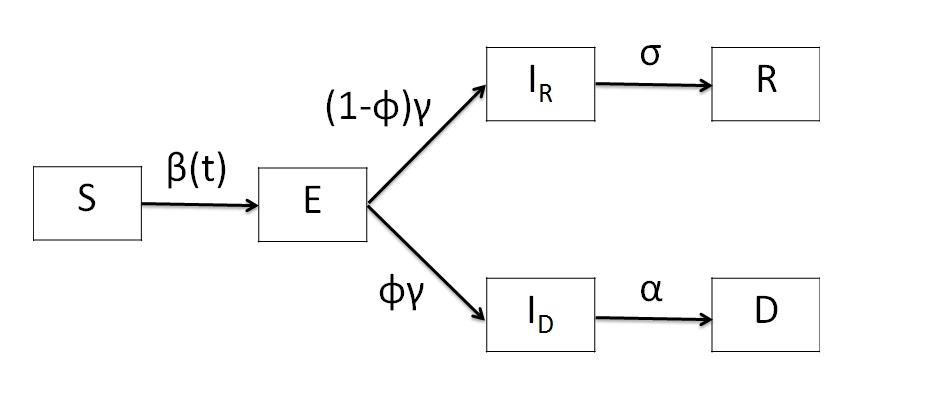

Figure S4: Flow diagram for the SEIR model with distinction between cases that survive and fatal cases.

Hence, we assume that individuals are born susceptible (S) to infection. Then, as time progresses they may become infected and move to the exposed compartment (E) at a time-dependent transmission rate $\beta(t)$. After the exposed stage, they become infectious and a proportion $1-\phi$, that will eventually recover, moves to the infectious $I_{R}$ compartment after a mean latent period $1/\gamma$. The proportion of fatal cases, $\phi$, moves to the $I_{D}$ compartment at the same rate. Individuals in the $I_{R}$ compartment recover after a mean infectious period $1/\sigma$. Lastly, $\alpha$ denotes the disease-related mortality rate.

This model can be expressed by the following set of ordinary differential equations (ODEs):

$$\frac{dS\left( t \right)}{dt}=-\beta\left( t \right)S\left( t \right)\frac{(I_{R}\left( t \right)+I_{D}\left( t \right))}{N\left( t \right)}$$

$$\frac{dE(t)}{dt}=\beta\left( t \right)S\left( t \right)\frac{(I_{R}\left( t \right)+I_{D}\left( t \right))}{N\left( t \right)}- \gamma E(t)$$

$$\frac{dI_{R}(t)}{dt}=(1-\phi)\gamma E\left( t \right)-\sigma I_{R}\left( t \right)$$

$$\frac{dI_{D}(t)}{dt}=\phi\gamma E\left( t \right)-\alpha I_{D}\left( t \right)$$

$$\frac{dR(t)}{dt}=\sigma I_{R}(t)$$

$$\frac{dD(t)}{dt}=\alpha I_{D}(t)$$

In this notation $N\left( t \right)=S\left( t \right)+E\left( t \right)+I_{R}\left( t \right)+I_{D}(t)+R(t)$ denotes population size.

The initial conditions at time $t=0$are given by $R\left( 0 \right)=0$, $I_{R}\left( 0 \right)=I_{D}\left( 0 \right)=0,$ $E(0)$ is an unknown parameter which is estimated from the data and $S\left( 0 \right)=N\left( 0 \right)-E(0)$where $N\left( 0 \right)$ is the population size at the start of the epidemic.

The effective reproduction number, denoted by $R_{e}\left( t \right)$, is defined as the number of secondary cases produced by a primary case during his/her infectious period. When $R_{e}\left( t \right)<1$ the epidemic will fade out, whereas, $R_{e}\left( t \right)>1$ indicates a persistent epidemic. The expression for $R_{e}\left( t \right)$ for this model is given below (see e.g. [2] for a resume explaining how to do the calculations).

$$R_{e}\left( t \right)=\beta\left( t \right)\left( \frac{\phi}{\sigma}+\frac{\left( 1-\phi\right)}{\alpha} \right)$$

**Monotonization data**

The data consists of cumulative number of (suspected, probable and confirmed) cases and deaths. Hence, this data is expected to increase monotonically over time. However, due to reclassification of suspected cases over time, the cumulative number of cases decreases at certain time points, resulting in negative number of new cases. We therefore applied the pooled adjacent violator algorithm (PAVA) algorithm to monotonize the cumulative data.

Denote the cumulative number of cases by$c_{i}, i=1,\ldots,n$. Suppose that $i^{*}$ is the first index for which$c_{i^{*}}<c_{i^{*}-1}$, i.e. the first index for which the monotone behavior is violated. The PAVA now states that these values need to be “pooled”. Hence, $c_{i^{*}}$ and $c_{i^{*}-1}$ are both replaced by

$$\frac{c_{i^{*}}+c_{i^{*}-1}}{2}.$$

The algorithm then proceeds by recursively checking monotone behavior and by pooling if necessary and stops when monotonicity is achieved.

**Estimation**

Fitting the ODE is done taking into account the specific reporting of cases and deaths. Reporting occurs at varying time intervals. Figure S4 schematically shows the reporting scheme of cases. The reporting scheme for deaths is similar but the dates at which reporting occurs are not necessarily the same.


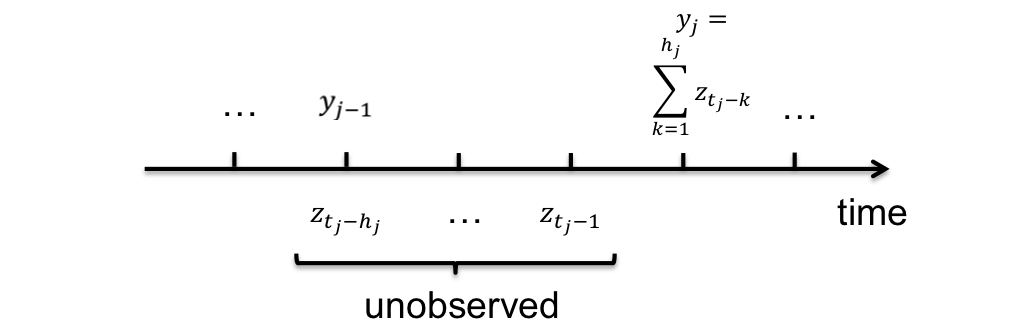


Figure S5 Schematic representation of reporting of case notifications.

We assume

$$y_{j} \sim NegBin\left( \rho\times{(I}_{\mathrm{new}}\left( t_{j}-1 \right)-I_{\mathrm{new}}(t_{j}-h_{j}-1)),\phi_{1} \right),$$

$d_{j} \sim NegBin\left( \rho\times(m\left( t_{j}-1 \right)-m\left( t_{j}-h_{j}- 1 \right)),\phi_{2} \right)$,

where $I_{\mathrm{new}}(t)$ ($m(t)$) is the expected cumulative number of cases (deaths) at time t obtained by solving $dI_{\mathrm{new}}(t)/dt=\gamma E\left( t \right)$ ($dm(t)/dt=\alpha I_{(D)}\left( t \right)$), $\rho$is the expected fraction of reported cases (deaths) and $\phi_{i}; i=1,2$ are overdispersion parameters. The objective function is then given by the sum of the negative-binomial loglikelihoods specified above.

Further, we model $R_{e}(t)$ as a piecewise constant function $R_{e(i)}$ as follows:

$${R_{e(0)}=R_{0}, R}_{e(i)}=R_{0}+r_{1}+\ldots+r_{i}; i=1,\ldots,n$$

Such that $r_{i}$ is the change in reproduction number compared to the previous time interval. This implies that $\beta_{i}=R_{e(i)}/\left( \frac{\phi}{\sigma}+\frac{\left( 1-\phi\right)}{\alpha} \right); i=0,\ldots,n$ is also piecewise constant. The length of the intervals is chosen to be 21 days.

Prior estimates for the latent period (9.4 days), the infectious period for survivors (16.4 days) and deceased (7.5 days) are used following Lewnard et al. [3]. The remaining parameters $\left( \phi_{1},\phi_{2}, E\left( 0 \right),\phi,\rho,R_{0},r_{i} \right);i=1,\ldots,n$ are estimated via Markov Chain Monte Carlo using the adaptive-mixture metropolis algorithm. We conducted 2,500,000 iterations retaining every 500^th^ iteration. Burn-in is based on the BMK convergence diagnostic. The univariate prior distributions are given in Table S1. Of these, the prior distributions for $\phi_{1},\phi_{2},E\left( 0 \right),R_{0}$ and $r_{i}$ are uninformative. The underreporting rate $\rho$ is assumed to follow a truncated normal distribution with mean 0.33 based on [4] and the case fatality ratio $\phi$ follows a beta distribution with mean 0.5.

| **Parameter** | **Definition** | **Prior distribution** |
| --- | --- | --- |
| $\phi_{1}$ | Overdispersion parameter cases | $HC(\alpha=25)$ |
| $\phi_{2}$ | Overdispersion parameter deaths | $HC(\alpha=25)$ |
| $E(0)$ | Number of exposed individuals at time 0 | $U(0,1)$ |
| $\phi$ | Case fatality ratio | $Beta(\alpha=10,\beta=10)$ |
| $\rho$ | Underreporting rate | $N(\mu=\frac{1}{3},\delta=0.1)$; truncated(0,1) |
| $R_{0}$ | Reproduction number 1st time period | $U(0,10)$ |
| $r_{i}$ | Changes in reproduction number | $U(-2,2)$ |

Table S1: Prior distributions.

**Parameter estimates**

The parameter estimates corresponding to Figure 3 and 4 for Forecariah, Conakry, Western Area Urban, and Grand Cape Mount are given in Table S2.

| **District** | $\hat{\boldsymbol{\phi}_{\boldsymbol{1}}}$ | $\hat{\boldsymbol{\phi}_{\boldsymbol{2}}}$ | $\hat{\boldsymbol{E(0)}}$ | $\hat{\boldsymbol{\phi}}$ | $\hat{\boldsymbol{\rho}}$ |
| --- | --- | --- | --- | --- | --- |
| Forecariah | 0.76 [0.54, 1.08] | 3.18 [1.70, 7.19] | 0.44 [0.07, 0.96] | 0.66 [0.54, 0.77] | 0.33 [0.13, 0.53] |
| Conakry | 0.62 [0.44, 0.89] | 1.61 [1.03, 2.60] | 61.4 [20.6, 97.7] | 0.53 [0.41, 0.67] | 0.34 [0.17, 0.54] |
| Western  Area Urb | 2.34 [1.71, 3.20] | 4.17 [2.31, 8.21] | 0.55 [0.10, 0.98] | 0.19 [0.16, 0.22] | 0.35 [0.16, 0.55] |
| Grand Cape  Mount | 0.72 [0.51, 1.00] | 0.62 [0.43, 0.90] | 0.54 [0.08, 0.98] | 0.62 [0.48, 0.77] | 0.33 [0.10, 0.54] |

Table S2: Parameter estimates with 95% posterior confidence intervals. Note that for Conakry a $\boldsymbol{U}\mathbf{(0,100)}$ prior for E(0) was used.

**Sensitivity analysis**

In the model that was used a number of assumptions were made. To assess the sensitivity of our results to these assumptions, the following models were fitted to the data of Nzerekore, Guinea.

Model 1: the final model: using the prior distributions specified in Table S1 and fixing the latent period and both infectious periods.

Models 2 to 5 look at the effect of the parameter $E\left( 0 \right)$, the number of exposed individuals on 23 May, 2014.

Models 2a – 2f: fixing $E\left( 0 \right)$ and varying its value from 0.01 to 10

Model 3: estimating $E\left( 0 \right)$ with uninformative prior $U(0,1000)$

Model 4: estimating $E\left( 0 \right)$ with uninformative prior $U\left( 0,1000 \right)$ and fixing $R_{0}$ to 2.00

Model 5: estimating $E\left( 0 \right)$ with uninformative prior $U(0,1000)$ and fixing $\rho$ to 0.33

In models 6 to 9 we look at the estimation of several fixed parameters.

Model 6: fixing $E\left( 0 \right)$ and estimating latent period

Model 7: fixing $E\left( 0 \right)$and estimating infectious period non-fatal cases

Model 8: fixing $E\left( 0 \right)$ and estimating infectious period fatal cases

Model 9: fixing $E\left( 0 \right)$ and estimating underreporting of deaths $\rho_{deaths}$ separately

In Models 10 to 13c we take into account that EVD can be transmitted through contact with the bodies of dead people. This model is expressed in the following set of differential equations.

$$\frac{dS\left( t \right)}{dt}=-\beta\left( t \right)S\left( t \right)\frac{(I_{R}\left( t \right)+I_{D}\left( t \right)+mD_{I}(t))}{N\left( t \right)}$$

$$\frac{dE(t)}{dt}=\beta\left( t \right)S\left( t \right)\frac{(I_{R}\left( t \right)+I_{D}\left( t \right)+m(t))}{N\left( t \right)}- \gamma E(t)$$

$$\frac{dI_{R}(t)}{dt}=(1-\phi)\gamma E\left( t \right)-\sigma I_{R}\left( t \right)$$

$$\frac{dI_{D}(t)}{dt}=\phi\gamma E\left( t \right)-\alpha I_{D}\left( t \right)$$

$$\frac{dR(t)}{dt}=\sigma I_{R}(t)$$

$$\frac{dD_{I}\left( t \right)}{dt}=\alpha I_{D}\left( t \right)-\kappa D_{I}\left( t \right)$$

$$\frac{dD_{R}\left( t \right)}{dt}=\kappa D_{I}(t)$$

Hence, when an individual dies from EVD, the body of that individual can transmit the disease (state $D_{I})$ for a period of time ($1/\kappa$) with transmission rate$m\beta(t)$. It then moves to state $D_{R}$ where transmission is no longer possible e.g. after burial.

Model 10: uninformative prior $U(0,20)$ for $m$ and Gamma distribution with mean 2 days and standard deviation 1.5 days for$1/\kappa$.

Models 11a-11b: fixing $m=1 \mathrm{and} 2$

Model 12: fixing $\frac{1}{\kappa}=2$ days

Models 13a-13c: fixing $\frac{1}{\kappa}=2$ days and $m=0.1, 0.5 \mathrm{and} 1$

Finally, since there is evidence of asymptomatic Ebola infections [Bellan et al. 2014], we assess the effect of protective immunity by asymptomatic infections in Models 14a-14d. These correspond to the following set of ODEs.

$$\frac{dS\left( t \right)}{dt}=-\beta\left( t \right)S\left( t \right)\frac{(I_{R}\left( t \right)+I_{D}\left( t \right))}{N\left( t \right)}$$

$$\frac{dE(t)}{dt}=\beta\left( t \right)S\left( t \right)\frac{(I_{R}\left( t \right)+I_{D}\left( t \right))}{N\left( t \right)}- \gamma E(t)$$

$$\frac{dI_{R}(t)}{dt}=(1-p)(1-\phi)\gamma E\left( t \right)-\sigma I_{R}\left( t \right)$$

$$\frac{dI_{D}(t)}{dt}=(1-p)\phi\gamma E\left( t \right)-\alpha I_{D}\left( t \right)$$

$$\frac{dR(t)}{dt}=\sigma I_{R}\left( t \right)+p\gamma E(t)$$

$$\frac{dD(t)}{dt}=\alpha I_{D}(t)$$

Where $p$ is the proportion of asymptomatic cases.

Models 14a-14e: fixing $p=0.1, 0.2, 0.3, 0.4 \mathrm{and} 0.45$

The results are given in Table S3 and S4. Looking at the DIC values of model 2, we see that there are very little differences, indicating that $E(0)$ is not estimable from the data. However, for large values of $E(0)$ (see model 2f) optimization leads to a local maximum with a very small reporting rate and high values of$R_{e}$, which are deemed implausible. Moreover, mixing in this model is very poor and convergence is not attained. The same is observed when estimating $E(0)$ with an uninformative prior (model 3), even when $R_{0}$ is kept constant (model 4). In model 5 the underreporting rate is fixed, this leads to convergence and good results, however, there is no improvement in DIC compared to model 1. For this reason, we chose to estimate $E(0)$ between 0 and 1 in our final model. Note that the value of $E(0)$ in the converged models only affects the estimates of $R_{e}$ in the first time periods. Making the most recent estimates robust to changes in$E(0)$.

In models 6, 7 and 8 the latent period and infectious periods are estimated, but again this leads to bad convergence and DIC does not improve. In model 9 we explored whether a different underreporting rate for deaths could be estimated. But bad mixing and high autocorrelation for that parameter indicated that this is not possible. Again, the most recent estimates of $R_{e}$ are quite robust in converged models.

In models 10 to 13c we look at the transmission of dead bodies. When estimating both parameters ($m \mathrm{and} \kappa)$, $m$ is estimated to be 0 and the model does not converge. The same result is obtained when fixing $1/\kappa$ to 2 days (model 12). Hence, $m$ is not estimable from the data. We therefore fix $m$ to different values both estimating and fixing $\kappa$ (models 11 and 13), but this does not lead to improvement in DIC or large changes in recent estimates of$R_{e}$.

Finally in models 14a-14d, we do see an improvement in DIC with growing proportion of asymptomatic cases, suggesting that taking into account the possibility of asymptomatic cases is coherent with observations.

| Model | 1 | 2a | 2b | 2c | 2d | 2e | 2f | 3 | 4 | 5 | 6 | 7 | 8 | 9 |
| --- | --- | --- | --- | --- | --- | --- | --- | --- | --- | --- | --- | --- | --- | --- |
| E(0) | 0.21 | **0.01** | **0.1** | **0.2** | **0.3** | **0.5** | **10** | 46.74 | 310.32 | 0.45 | **0.28** | **0.28** | **0.28** | **0.1** |
| $1/\gamma$ | **9.4** | **9.4** | **9.4** | **9.4** | **9.4** | **9.4** | **9.4** | **9.4** | **9.4** | **9.4** | 1.92 | **9.4** | **9.4** | **9.4** |
| $1/\sigma$ | **16.4** | **16.4** | **16.4** | **16.4** | **16.4** | **16.4** | **16.4** | **16.4** | **16.4** | **16.4** | **16.4** | 10.17 | **16.4** | **16.4** |
| $1/\alpha$ | **7.5** | **7.5** | **7.5** | **7.5** | **7.5** | **7.5** | **7.5** | **7.5** | **7.5** | **7.5** | **7.5** | **7.5** | 1.03 | **7.5** |
| $\phi$ | 0.58 | 0.60 | 0.58 | 0.58 | 0.58 | 0.58 | 0.56 | 0.56 | 0.57 | 0.59 | 0.53 | 0.54 | 0.57 | 0.37 |
| $\rho$ | 0.32 | 0.36 | 0.33 | 0.32 | 0.32 | 0.31 | 0.0009 | 0.0009 | 0.0010 | **0.33** | 0.0009 | 0.0009 | 0.32 | 0.43 |
| $\rho_{deaths}$ | - | - | - | - | - | - | - | - | - | - | - | - | - | 1.00 |
| $R_{e(0)}$ | 2.64 | 3.73 | 2.88 | 2.61 | 2.47 | 2.29 | 3.39 | 2.49 | **2.00** | 2.29 | 2.92 | 4.17 | 2.60 | 2.85 |
| $R_{e(1)}$ | 2.23 | 2.29 | 2.12 | 2.20 | 2.25 | 2.32 | 2.41 | 2.54 | 2.70 | 2.33 | 1.58 | 2.60 | 2.57 | 2.33 |
| $R_{e(2)}$ | 1.94 | 1.79 | 1.96 | 1.96 | 1.98 | 1.98 | 2.41 | 2.40 | 2.32 | 1.96 | 2.08 | 2.04 | 2.12 | 2.00 |
| $R_{e(3)}$ | 1.02 | 0.98 | 1.03 | 1.03 | 1.03 | 1.02 | 1.87 | 1.89 | 168 | 1.04 | 1.59 | 1.95 | 1.10 | 1.04 |
| $R_{e(4)}$ | 0.60 | 0.63 | 0.61 | 0.60 | 0.60 | 0.59 | 2.08 | 2.10 | 1.75 | 0.60 | 2.28 | 2.40 | 0.67 | 0.62 |
| $R_{e(5)}$ | 0.37 | 0.35 | 0.36 | 0.37 | 0.37 | 0.38 | 2.20 | 2.28 | 1.75 | 0.37 | 2.42 | 2.74 | 0.46 | 0.36 |
| $R_{e(6)}$ | 0.26 | 0.27 | 0.27 | 0.25 | 0.26 | 0.26 | 2.17 | 2.22 | 1.66 | 0.26 | 3.09 | 2.86 | 0.24 | 0.26 |
| $R_{e(7)}$ | 0.24 | 0.23 | 0.24 | 0.24 | 0.24 | 0.24 | 1.91 | 2.03 | 1.23 | 0.24 | 2.75 | 2.86 | 0.16 | 0.24 |
| $R_{e(8)}$ | 0.38 | 0.40 | 0.41 | 0.43 | 0.40 | 0.40 | 1.94 | 2.01 | 1.48 | 0.43 | 2.60 | 2.78 | 0.44 | 0.41 |
| DIC | 457.57 | 459.81 | 456.77 | 457.13 | 456.43 | 456.66 | 456.37 | 454.24 | 454.65 | 458.66 | 581.37 | 463.72 | 457.25 | 458.19 |

Table S3: Parameter estimates sensitivity analysis. Fixed values are indicated in bold,
blue values indicate model differences compared to the final model 1.

| Model | 10 | 11a | 11b | 12 | 13a | 13b | 13c | 14a | 14b | 14c | 14d | 14e |
| --- | --- | --- | --- | --- | --- | --- | --- | --- | --- | --- | --- | --- |
| E(0) | 0.20 | 0.20 | 0.20 | 0.49 | 0.21 | 0.19 | 0.71 | 0.23 | 0.26 | 0.29 | 0.32 | 0.36 |
| $1/\gamma$ | **9.4** | **9.4** | **9.4** | **9.4** | **9.4** | **9.4** | **9.4** | **9.4** | **9.4** | **9.4** | **9.4** | **9.4** |
| $1/\sigma$ | **16.4** | **16.4** | **16.4** | **16.4** | **16.4** | **16.4** | **16.4** | **16.4** | **16.4** | **16.4** | **16.4** | **16.4** |
| $1/\alpha$ | **7.5** | **7.5** | **7.5** | **7.5** | **7.5** | **7.5** | **7.5** | **7.5** | **7.5** | **7.5** | **7.5** | **7.5** |
| $p$ | - | - | - | **-** | **-** | **-** | **-** | **0.1** | **0.2** | **0.3** | **0.4** | **0.45** |
| $1/\kappa$ | 0.67 | 0.56 | 0.56 | **2.00** | **2.00** | **2.00** | **2.00** | **-** | **-** | - | **-** | **-** |
| $m$ | 0.00 | **1.00** | **2.00** | 0.00 | **0.10** | **0.50** | **1.00** | **-** | **-** | - | **-** | **-** |
| $\phi$ | 0.58 | 0.58 | 0.57 | 0.57 | 0.60 | 0.57 | 0.55 | 0.58 | 0.58 | 0.59 | 0.59 | 0.59 |
| $\rho$ | 0.33 | 0.32 | 0.32 | 0.001 | 0.32 | 0.32 | 0.0009 | 0.32 | 0.32 | 0.32 | 0.33 | 0.33 |
| $R_{e(0)}$ | 2.65 | 2.60 | 2.57 | 3.93 | 2.61 | 2.56 | 4.22 | 2.95 | 3.32 | 3.76 | 4.41 | 4.81 |
| $R_{e(1)}$ | 2.22 | 2.15 | 2.13 | 2.73 | 2.18 | 2.10 | 2.66 | 2.49 | 2.84 | 3.24 | 3.78 | 4.08 |
| $R_{e(2)}$ | 1.93 | 1.91 | 1.87 | 2.10 | 1.92 | 1.85 | 2.08 | 2.13 | 2.35 | 2.63 | 2.98 | 3.23 |
| $R_{e(3)}$ | 1.03 | 0.99 | 0.98 | 1.51 | 1.01 | 0.95 | 1.89 | 1.17 | 1.35 | 1.56 | 1.85 | 2.06 |
| $R_{e(4)}$ | 0.60 | 0.60 | 0.59 | 1.29 | 0.59 | 0.58 | 2.27 | 0.66 | 0.74 | 0.84 | 1.01 | 1.12 |
| $R_{e(5)}$ | 0.37 | 0.36 | 0.36 | 0.96 | 0.37 | 0.36 | 2.62 | 0.43 | 0.48 | 0.54 | 0.62 | 0.68 |
| $R_{e(6)}$ | 0.27 | 0.25 | 0.25 | 0.80 | 0.25 | 0.24 | 2.77 | 0.28 | 0.31 | 0.37 | 0.43 | 0.48 |
| $R_{e(7)}$ | 0.23 | 0.24 | 0.23 | 0.69 | 0.23 | 0.23 | 2.71 | 0.28 | 0.32 | 0.35 | 0.42 | 0.46 |
| $R_{e(8)}$ | 0.44 | 0.40 | 0.39 | 1.20 | 0.41 | 0.44 | 2.68 | 0.43 | 0.43 | 0.41 | 0.48 | 0.45 |
| DIC | 460.04 | 463.74 | 460.34 | 462.43 | 457.02 | 463.52 | 460.75 | 456.32 | 456.40 | 455.31 | 454.79 | 453.52 |

Table S4: Parameter estimates sensitivity analysis. Fixed values are indicated in bold,
blue values indicate changes compared to the final model 1.

**Software**

The MCMC procedure is performed in R 3.1.1 using the LaplacesDemon package [1]. Our code is made publicly available at www.simid.be.

**References**

1. Hall B. LaplacesDemon: An R Package for Bayesian Inference [Internet].Statisticat, LLC. Available from: http://www.icesi.edu.co/CRAN/web/packages/LaplacesDemon/vignettes/LaplacesDemonTutorial.pdf.

2. Mathematical and Statistical Estimation Approaches in Epidemiology / Chowell, G. [edit.]; et al. - ISBN 978-90-481-2312-4 - Springer Netherlands, 2009

3. Lewnard JA, Ndeffo Mbah ML, Alfaro-Murillo JA, Altice FL, Bawo L, Nyenswah TG, Galvani AP. Dynamics and control of Ebola virus transmission in Montserrado, Liberia: a mathematical modelling analysis. The Lancet Infectious Diseases. 2014

4. http://www.cdc.gov/vhf/ebola/outbreaks/2014-west-africa/qa-mmwr-estimating-future-cases.html
