# Supplementary material for: Spatiotemporal Evolution of Ebola Virus Disease at Sub-National Level during the 2014 West Africa Epidemic: Model Scrutiny and Data Meagreness
Source: PLoS One. 2016 Jan 15;11(1):e0147172. doi: 10.1371/journal.pone.0147172 (PMC4714854; doi:10.1371/journal.pone.0147172)
Supplement: S1 Table — (PDF) [file pone.0147172.s007.pdf]

Parameter	Definition	Prior distribution
ϕ_1	Overdispersion parameter cases	$\mathrm{HC}(\alpha = 25)$
ϕ_2	Overdispersion parameter deaths	$\mathrm{HC}(\alpha = 25)$
$\mathrm{E}(0)$	Number of exposed individuals at time 0	$U(0,1)$
ϕ	Case fatality ratio	$\mathrm{Beta}(\alpha = 10, \beta = 10)$
ρ	Underreporting rate	$\mathrm{N}(\mu = \frac{1}{3}, \delta = 0.1)$; truncated(0,1)
R_0	Reproduction number 1st time period	$\mathrm{U}(0,10)$
r_i	Changes in reproduction number	$\mathrm{U}(-2,2)$

Table S1: Prior distributions.
