# Supplementary material for: Spatiotemporal Evolution of Ebola Virus Disease at Sub-National Level during the 2014 West Africa Epidemic: Model Scrutiny and Data Meagreness
Source: PLoS One. 2016 Jan 15;11(1):e0147172. doi: 10.1371/journal.pone.0147172 (PMC4714854; doi:10.1371/journal.pone.0147172)
Supplement: S2 Table — Note that for Conakry a U(0, 100) prior for E(0) was used. (PDF) [file pone.0147172.s008.pdf]

District	$\widehat{\phi_1}$	$\widehat{\phi_2}$	$\widehat{E(0)}$	$\widehat{\phi}$	$\widehat{\rho}$
Forecariah	0.76 [0.54, 1.08]	3.18 [1.70, 7.19]	0.44 [0.07, 0.96]	0.66 [0.54, 0.77]	0.33 [0.13, 0.53]
Conakry	0.62 [0.44, 0.89]	1.61 [1.03, 2.60]	61.4 [20.6, 97.7]	0.53 [0.41, 0.67]	0.34 [0.17, 0.54]
Western Area Urb	2.34 [1.71, 3.20]	4.17 [2.31, 8.21]	0.55 [0.10, 0.98]	0.19 [0.16, 0.22]	0.35 [0.16, 0.55]
Grand Cape Mount	0.72 [0.51, 1.00]	0.62 [0.43, 0.90]	0.54 [0.08, 0.98]	0.62 [0.48, 0.77]	0.33 [0.10, 0.54]

Table S2: Parameter estimates with 95% posterior confidence intervals. Note that for Conakry a $\boldsymbol{U(0, 100)}$ prior for E(0) was used.
