# Supplementary material for: Spatiotemporal Evolution of Ebola Virus Disease at Sub-National Level during the 2014 West Africa Epidemic: Model Scrutiny and Data Meagreness
Source: PLoS One. 2016 Jan 15;11(1):e0147172. doi: 10.1371/journal.pone.0147172 (PMC4714854; doi:10.1371/journal.pone.0147172)
Supplement: S3 Table — Fixed values are indicated in bold, blue values indicate model differences compared to the final model 1. (PDF) [file pone.0147172.s009.pdf]

Model	1	2a	2b	2c	2d	2e	2f	3	4	5	6	7	8	9
E(0)	0.21	**0.01**	**0.1**	**0.2**	**0.3**	**0.5**	**10**	46.74	310.32	0.45	**0.28**	**0.28**	**0.28**	**0.1**
$1/\gamma$	**9.4**	**9.4**	**9.4**	**9.4**	**9.4**	**9.4**	**9.4**	**9.4**	**9.4**	**9.4**	1.92	**9.4**	**9.4**	**9.4**
$1/\sigma$	**16.4**	**16.4**	**16.4**	**16.4**	**16.4**	**16.4**	**16.4**	**16.4**	**16.4**	**16.4**	**16.4**	10.17	**16.4**	**16.4**
$1/\alpha$	**7.5**	**7.5**	**7.5**	**7.5**	**7.5**	**7.5**	**7.5**	**7.5**	**7.5**	**7.5**	**7.5**	**7.5**	1.03	**7.5**
ϕ	0.58	0.60	0.58	0.58	0.58	0.58	0.56	0.56	0.57	0.59	0.53	0.54	0.57	0.37
ρ	0.32	0.36	0.33	0.32	0.32	0.31	0.0009	0.0009	0.0010	**0.33**	0.0009	0.0009	0.32	0.43
ρ_{deaths}	-	-	-	-	-	-	-	-	-	-	-	-	-	1.00
$R_{e(0)}$	2.64	3.73	2.88	2.61	2.47	2.29	3.39	2.49	**2.00**	2.29	2.92	4.17	2.60	2.85
$R_{e(1)}$	2.23	2.29	2.12	2.20	2.25	2.32	2.41	2.54	2.70	2.33	1.58	2.60	2.57	2.33
$R_{e(2)}$	1.94	1.79	1.96	1.96	1.98	1.98	2.41	2.40	2.32	1.96	2.08	2.04	2.12	2.00
$R_{e(3)}$	1.02	0.98	1.03	1.03	1.03	1.02	1.87	1.89	168	1.04	1.59	1.95	1.10	1.04
$R_{e(4)}$	0.60	0.63	0.61	0.60	0.60	0.59	2.08	2.10	1.75	0.60	2.28	2.40	0.67	0.62
$R_{e(5)}$	0.37	0.35	0.36	0.37	0.37	0.38	2.20	2.28	1.75	0.37	2.42	2.74	0.46	0.36
$R_{e(6)}$	0.26	0.27	0.27	0.25	0.26	0.26	2.17	2.22	1.66	0.26	3.09	2.86	0.24	0.26
$R_{e(7)}$	0.24	0.23	0.24	0.24	0.24	0.24	1.91	2.03	1.23	0.24	2.75	2.86	0.16	0.24
$R_{e(8)}$	0.38	0.40	0.41	0.43	0.40	0.40	1.94	2.01	1.48	0.43	2.60	2.78	0.44	0.41
DIC	457.57	459.81	456.77	457.13	456.43	456.66	456.37	454.24	454.65	458.66	581.37	463.72	457.25	458.19

Table S3: Parameter estimates sensitivity analysis. Fixed values are indicated in bold, blue values indicate model differences compared to the final model 1.
