# Supplementary material for: Spatiotemporal Evolution of Ebola Virus Disease at Sub-National Level during the 2014 West Africa Epidemic: Model Scrutiny and Data Meagreness
Source: PLoS One. 2016 Jan 15;11(1):e0147172. doi: 10.1371/journal.pone.0147172 (PMC4714854; doi:10.1371/journal.pone.0147172)
Supplement: S4 Table — Fixed values are indicated in bold, blue values indicate changes compared to the final model 1. (PDF) [file pone.0147172.s010.pdf]

Model	10	11a	11b	12	13a	13b	13c	14a	14b	14c	14d	14e
E(0)	0.20	0.20	0.20	0.49	0.21	0.19	0.71	0.23	0.26	0.29	0.32	0.36
$1/\gamma$	**9.4**	**9.4**	**9.4**	**9.4**	**9.4**	**9.4**	**9.4**	**9.4**	**9.4**	**9.4**	**9.4**	**9.4**
$1/\sigma$	**16.4**	**16.4**	**16.4**	**16.4**	**16.4**	**16.4**	**16.4**	**16.4**	**16.4**	**16.4**	**16.4**	**16.4**
$1/\alpha$	**7.5**	**7.5**	**7.5**	**7.5**	**7.5**	**7.5**	**7.5**	**7.5**	**7.5**	**7.5**	**7.5**	**7.5**
p	-	-	-	**-**	**-**	**-**	**-**	**0.1**	**0.2**	**0.3**	**0.4**	**0.45**
$1/\kappa$	0.67	0.56	0.56	**2.00**	**2.00**	**2.00**	**2.00**	**-**	**-**	-	**-**	**-**
m	0.00	**1.00**	**2.00**	0.00	**0.10**	**0.50**	**1.00**	**-**	**-**	-	**-**	**-**
ϕ	0.58	0.58	0.57	0.57	0.60	0.57	0.55	0.58	0.58	0.59	0.59	0.59
ρ	0.33	0.32	0.32	0.001	0.32	0.32	0.0009	0.32	0.32	0.32	0.33	0.33
$R_{e(0)}$	2.65	2.60	2.57	3.93	2.61	2.56	4.22	2.95	3.32	3.76	4.41	4.81
$R_{e(1)}$	2.22	2.15	2.13	2.73	2.18	2.10	2.66	2.49	2.84	3.24	3.78	4.08
$R_{e(2)}$	1.93	1.91	1.87	2.10	1.92	1.85	2.08	2.13	2.35	2.63	2.98	3.23
$R_{e(3)}$	1.03	0.99	0.98	1.51	1.01	0.95	1.89	1.17	1.35	1.56	1.85	2.06
$R_{e(4)}$	0.60	0.60	0.59	1.29	0.59	0.58	2.27	0.66	0.74	0.84	1.01	1.12
$R_{e(5)}$	0.37	0.36	0.36	0.96	0.37	0.36	2.62	0.43	0.48	0.54	0.62	0.68
$R_{e(6)}$	0.27	0.25	0.25	0.80	0.25	0.24	2.77	0.28	0.31	0.37	0.43	0.48
$R_{e(7)}$	0.23	0.24	0.23	0.69	0.23	0.23	2.71	0.28	0.32	0.35	0.42	0.46
$R_{e(8)}$	0.44	0.40	0.39	1.20	0.41	0.44	2.68	0.43	0.43	0.41	0.48	0.45
DIC	460.04	463.74	460.34	462.43	457.02	463.52	460.75	456.32	456.40	455.31	454.79	453.52

Table S4: Parameter estimates sensitivity analysis. Fixed values are indicated in bold, blue values indicate changes compared to the final model 1.
